# Supplementary material for: Analysis of PPARγ Signaling Activity in Psoriasis
Source: Int J Mol Sci. 2021 Aug 10;22(16):8603. doi: 10.3390/ijms22168603 (PMC8395241; doi:10.3390/ijms22168603)
Supplement: Supplementary file 1 [file ijms-22-08603-s001.zip › Supplemental materials_Analysis of PPARg signaling activity in psoriasis/Pathway models/Models images and html files/Interleukin-17 and interleukin-22 signaling in psoriasis/7518.html]

MMP1


# Protein MMP1

|  |  |
| --- | --- |
| URN | urn:agi-llid:4312 |
| Total Entities | 0 |
| Connectivity | 7276 |
| Name | MMP1 |
| Description | matrix metallopeptidase 1 |
| Notes | This gene encodes a member of the peptidase M10 family of matrix metalloproteinases (MMPs). Proteins in this family are involved in the breakdown of extracellular matrix in normal physiological processes, such as embryonic development, reproduction, and tissue remodeling, as well as in disease processes, such as arthritis and metastasis. The encoded preproprotein is proteolytically processed to generate the mature protease. This secreted protease breaks down the interstitial collagens, including types I, II, and III. The gene is part of a cluster of MMP genes on chromosome 11. Mutations in this gene are associated with chronic obstructive pulmonary disease (COPD). Alternative splicing results in multiple transcript variants, at least one of which encodes an isoform that is proteolytically processed. [provided by RefSeq, Jan 2016] |
| Primary Cell Localization | Extracellular |

---

|  |  |
| --- | --- |
| Pathway | Osteoarthritis |
|  | Skin Aging |
|  | Plasmin Effects in Inflammation |
|  | Bradykinin Effects in Inflammation |
|  | Nociception Expression Targets Signaling |
|  | CCR1 Expression Targets |
|  | Scavenger Receptor OLR1 in Inflammation Related Endothelial Dysfunction |
|  | Proteins Involved in Dilated Cardiomyopathy |
|  | Proteins Involved in Arterial Hypertension |
|  | Myocardial Remodeling |
|  | Myocardial Infarction |
|  | Proteins with Altered Expression in Melanoma |
|  | Melanoma |
|  | Proteins Involved in Melanoma |
|  | IL17 Signaling in Psoriasis |
|  | IL22 Induces Keratinocyte Proliferation in Psoriasis |
|  | Proteins with Altered Expression in Psoriasis |
|  | TGF-beta Signaling in Skin Aging |
|  | Proteins Involved in Photoaging |
|  | Extracellular Matrix Degradation in Skin Aging |
|  | Proteins Involved in Colorectal Neoplasms |
|  | TNF and IL1B Induce Metalloproteinase Synthesis in Osteoarthritis |
|  | Proteins Involved in Osteoarthritis |
|  | Proteins Involved in Polycystic Ovary Syndrome |
|  | Synovial Fibroblast Activation in Psoriatic Arthritis |
|  | Keratinocyte Activation in Psoriatic Arthritis |
|  | Synovial Fibroblast Activation by Citokines in Rheumatoid Arthritis |
|  | TLR2 Induced Synovial Fibroblast Activation in Rheumatoid Arthritis |
|  | Proteins Involved in Rheumatoid Arthritis |
|  | Proteins Involved in Systemic Scleroderma |
|  | Skin Fibrosis |
|  | Cathepsins Role in Periodontitis |
|  | Proteins Involved in Periodontitis |
|  | Proteins Involved in Astrocytoma |
|  | Glioma Invasion Signaling |
|  | Proteins Involved in Glioma |
|  | Proteins Involved in Adhesion, Migration and Invasion of Endometriotic Cell |
|  | Proteins Involved in Endometriosis |
|  | Proteins with Altered Expression in Endometriosis |
|  | Proteins Involved in Age-Related Macular Degeneration |
|  | PGF2-alpha Effect on Treatment |
|  | Proteins Involved in Chronic Obstructive Pulmonary Disease |
|  | Alveolar Epithelial Cell Dysfunction in COPD |
|  | Noradrenaline/Gs Expression Targets |
|  | Serotonin/Gs Expression Targets |
|  | Noreadrenaline/Gq Expression Targets |
|  | Epinephrine/Gs Expression Targets |
|  | Acetylcholine Expression Targets |
|  | Serotonin/Gq Expression Targets |
|  | Epinephrine/Gq Expression Targets |
|  | Elevated Receptors -> Expression Targets in Heart |
|  | CXCL12 Expression Targets |
|  | CCL3 Expression Targets |
|  | CSF2 -> NF-kB Expression Targets |
|  | CSF2 -> STAT Expression Targets |
|  | LIF Expression Targets |
|  | EGF -> CTNN Expression Targets |
|  | EGF -> AP-1/ATF Expression Targets |
|  | EGF -> CREB/CREBBP/ELK/SRF/MYC Expression Targets |
|  | EGF -> MEF/MYOD/NFATC Expression Targets |
|  | EGF -> STAT Expression Targets |
|  | Erythropoietin -> NF-kB Expression Targets |
|  | Erythropoietin -> AP-1/MYC/CREB Expression Targets |
|  | Erythropoietin -> STAT Expression Targets |
|  | Erythropoietin -> ELK/SRF Expression Targets |
|  | F2 -> AP-1/CREB/ELK/SRF/SP1 Expression Targets |
|  | F2 -> STAT1/NF-kB Expression Targets |
|  | PLG -> AP-1/CREB/ELK/SRF/SP1 Expression Targets |
|  | PLG -> STAT1/NF-kB Expression Targets |
|  | FGF2 -> STAT Expression Targets |
|  | FGF1 -> AP-1/CREB/ELK/SRF/MYC Expression Targets |
|  | FGF1 -> STAT Expression Targets |
|  | FGF7 -> AP-1/CREB/CREBBP/MYC Expression Targets |
|  | FGF2 -> AP-1/CREB/CREBBP/ELK/SRF/MYC Expression Targets |
|  | HGF -> STAT Expression Targets |
|  | HGF -> AP-1/CREB/ELK/SRF/MYC Expression Targets |
|  | EGFR Transactivation in Cancer and Non-Cancer Cells |
|  | Proteins with Altered Expression in Cancer Metastases |
|  | BSG (CD147) in Cancer Cells Motility, Invasion and Survival |
|  | IGF1 -> CEBPA/FOXO1A Expression Targets |
|  | Insulin -> STAT Expression Targets |
|  | Insulin -> CEBPA/CTNNB/FOXA/FOXO Expression Targets |
|  | Insulin -> MEF/MYOD Expression Targets |
|  | Insulin -> ELK/SRF/HIF1A/MYC/SREBF Expression Targets |
|  | CTGF -> AP-1/CREB/MYC Expression Targets |
|  | IL6 Expression Targets |
|  | IL10 Expression Targets |
|  | IL22 Expression Targets |
|  | IL11 Expression Targets |
|  | Prostaglandin F Expression Targets |
|  | PAF/Gq -> AP-1/ATF1/CREB/ERK/SRF Expression Targets |
|  | PGE1 Expression Targets |
|  | CD40 -> Expression Targets in Thymus |
|  | Elevated Receptors -> Expression Targets in Muscle |
|  | OSM/OSMR Expression Targets |
|  | PDGFD -> AP-1 Expression Targets |
|  | PDGFD -> STAT Expression Targets |
|  | PDGF -> AP-1/CREB/CREBBP/MYC Expression Targets |
|  | PDGF -> STAT Expression Targets |
|  | OXT Expression Targets |
|  | POMC Expression Targets |
|  | EDN1 Expression Targets |
|  | AGT -> STAT Expression Targets |
|  | PRL/PRLR Expression Targets |
|  | PRL/GHR -> STAT Expression Targets |
|  | PRL/GHR -> NF/kB/ELK/SRF/MYC Expression Targets |
|  | PAF Expression Targets |
|  | TGFA -> STAT Expression Targets |
|  | TGFA -> CTNNB/CTNND Expression Targets |
|  | TGFA/AP-1/ATF Expression Targets |
|  | TGFA -> CREB/CREBBP/ELK-SRF/MYC Expression Targets |
|  | TGFB2-TGFBR1 Expression Targets |
|  | TGFB1-TGFBR1 Expression Targets |
|  | TGFB2-TGFBR2 Expression Targets |
|  | TGFB1-TGFBR2 Expression Targets |
|  | TGFB1-ACVRL1 Expression Targets |
|  | TNF -> NF-kB Expression Targets |
|  | TNF -> AP-1 Expression Targets |
|  | CD40LG -> STAT Expression Targets |
|  | TNF -> STAT Expression Targets |
|  | TLR4 -> AP-1 Expression Targets |
|  | TLR5 Expression Targets |
|  | IL1B -> NO Expression Targets |
|  | TLR1 -> 2/6 Expression Targets |
|  | IL1B Expression Targets |
|  | TLR4 -> NF-kB/IRF Expression Targets |
|  | IL1A Expression Targets |
|  | PLAU -> STAT1 Expression Targets |
|  | PLAU -> ELK-SRF/AP-1 Expression Targets |
|  | VEGFA -> ATF/CREB/ELK-SRF Expression Targets |
|  | VEGFA -> STAT Expression Targets |
|  | VEGFA -> AP-1/CREBBP/MYC Expression Targets |
|  | VEGFA -> CTNNB/CTNND Expression Targets |
|  | WNT5A Expression Targets |
|  | ace2 |
|  | pprarg neg, uknown targets, ps-positive |
|  | prarg neg,ukn expres targets, ps-positive |
|  | PPARG negative targets |
|  | pparg expr target\_regulators |
|  | Neighbors of disease exacerbation |
|  | Neighbors of tumor progression |
|  | Neighbors of cancer progression |
|  | Neighbors of overall survival |
|  | Neighbors of disease severity |
|  | Neighbors of tumor size |
|  | Neighbors of lesion size |
|  | Neighbors of mortality |
|  | Neighbors of survival time |
|  | Neighbors of functional recovery |
|  | Neighbors of cancer survival |
|  | Neighbors of clinical stage |
|  | Neighbors of lethality |
|  | Neighbors of recurrence-free survival |
|  | Neighbors of disease-free survival |
|  | Neighbors of treatment outcome |
|  | Neighbors of tumor recurrence |
|  | Neighbors of tumor promotion |
|  | Neighbors of morbidity |
|  | Neighbors of bone mineral density |
|  | Neighbors of adverse outcome |
|  | Neighbors of cell density |
|  | Neighbors of cancer-specific survival |
|  | Neighbors of shear stress |
|  | Neighbors of blood pressure |
|  | Neighbors of tumor grade |
|  | Neighbors of vascular reactivity |
|  | Neighbors of body mass index |
|  | Neighbors of cancer risk |
|  | Neighbors of cancer incidence |
|  | Neighbors of ejection fraction |
|  | Neighbors of vascular tone |
|  | Neighbors of left ventricular ejection fraction |
|  | Neighbors of breast cancer risk |
|  | Neighbors of pathological response |
|  | Neighbors of disease exacerbation |
|  | Neighbors of tumor progression |
|  | Neighbors of cancer progression |
|  | Neighbors of overall survival |
|  | Neighbors of disease severity |
|  | Neighbors of tumor size |
|  | Neighbors of lesion size |
|  | Neighbors of mortality |
|  | Neighbors of survival time |
|  | Neighbors of functional recovery |
|  | Neighbors of cancer survival |
|  | Neighbors of clinical stage |
|  | Neighbors of lethality |
|  | Neighbors of recurrence-free survival |
|  | Neighbors of disease-free survival |
|  | Neighbors of treatment outcome |
|  | Neighbors of tumor recurrence |
|  | Neighbors of tumor promotion |
|  | Neighbors of morbidity |
|  | Neighbors of bone mineral density |
|  | Neighbors of adverse outcome |
|  | Neighbors of cell density |
|  | Neighbors of cancer-specific survival |
|  | Neighbors of shear stress |
|  | Neighbors of blood pressure |
|  | Neighbors of tumor grade |
|  | Neighbors of vascular reactivity |
|  | Neighbors of body mass index |
|  | Neighbors of cancer risk |
|  | Neighbors of cancer incidence |
|  | Neighbors of ejection fraction |
|  | Neighbors of vascular tone |
|  | Neighbors of left ventricular ejection fraction |
|  | Neighbors of breast cancer risk |
|  | Neighbors of pathological response |
|  | Neighbors of immune response |
|  | Neighbors of inflammatory response |
|  | Neighbors of wound healing |
|  | Neighbors of angiogenesis |
|  | Neighbors of innate immune response |
|  | Neighbors of cell function |
|  | Neighbors of cell formation |
|  | Neighbors of osteoclast development |
|  | Neighbors of epithelial to mesenchymal transition |
|  | Neighbors of immunity |
|  | Neighbors of cell proliferative response |
|  | Neighbors of chemotaxis |
|  | Neighbors of cell survival |
|  | Neighbors of osteoclast differentiation |
|  | Neighbors of cell phenotype |
|  | Neighbors of T-cell proliferation |
|  | Neighbors of tissue remodeling |
|  | Neighbors of cell adhesion |
|  | Neighbors of endothelial cell proliferation |
|  | Neighbors of fibrogenesis |
|  | Neighbors of tumor growth |
|  | Neighbors of tissue repair |
|  | Neighbors of cell infiltration |
|  | Neighbors of cell interaction |
|  | Neighbors of SMC proliferation |
|  | Neighbors of lumen formation |
|  | Neighbors of endothelial cell migration |
|  | Neighbors of lipid storage |
|  | Neighbors of pregnancy |
|  | Neighbors of aging |
|  | Neighbors of cell motility |
|  | Neighbors of bone resorption |
|  | Neighbors of monocyte migration |
|  | Neighbors of fibroblast proliferation |
|  | Neighbors of adipocyte differentiation |
|  | Neighbors of regeneration |
|  | Neighbors of cell migration |
|  | Neighbors of cancer growth |
|  | Neighbors of stem cell proliferation |
|  | Neighbors of smooth muscle cell migration |
|  | Neighbors of hepatic regeneration |
|  | Neighbors of transendothelial migration |
|  | Neighbors of leukocyte migration |
|  | Neighbors of monocyte recruitment |
|  | Neighbors of cell invasion |
|  | Neighbors of heart function |
|  | Neighbors of bone remodeling |
|  | Neighbors of ossification |
|  | Neighbors of lung development |
|  | Neighbors of autophagy |
|  | Neighbors of transcription activation |
|  | Neighbors of senescence |
|  | Neighbors of hepatocyte proliferation |
|  | Neighbors of endothelial cell function |
|  | Neighbors of neuronal death |
|  | Neighbors of cell growth |
|  | Neighbors of ROS generation |
|  | Neighbors of epithelial cell proliferation |
|  | Neighbors of cancer cell growth |
|  | Neighbors of hemato-encephalic barrier |
|  | Neighbors of cellular senescence |
|  | Neighbors of colony formation |
|  | Neighbors of cell differentiation |
|  | Neighbors of stem cell migration |
|  | Neighbors of psoriasis |
|  | Neighbors of inflammatory disease |
|  | Neighbors of rheumatoid arthritis |
|  | Neighbors of atherosclerosis |
|  | Neighbors of fibrosis |
|  | Neighbors of colitis |
|  | Neighbors of arthritis |
|  | Neighbors of injury |
|  | Neighbors of atherogenesis |
|  | Neighbors of vascular remodeling |
|  | Neighbors of metastasis |
|  | Neighbors of carcinogenesis |
|  | Neighbors of multiple sclerosis |
|  | Neighbors of chronic inflammation |
|  | Neighbors of inflammation |
|  | Neighbors of tumor microenvironment |
|  | Neighbors of pathological angiogenesis |
|  | Neighbors of obesity |
|  | Neighbors of vascular disease |
|  | Neighbors of diabetes mellitus |
|  | Neighbors of asthma |
|  | Neighbors of hyperplasia |
|  | Neighbors of osteoarthritis |
|  | Neighbors of liver fibrosis |
|  | Neighbors of chronic obstructive pulmonary disease |
|  | Neighbors of myocardial infarction |
|  | Neighbors of leukocyte infiltration |
|  | Neighbors of reperfusion injury |
|  | Neighbors of airway inflammation |
|  | Neighbors of hepatitis |
|  | Neighbors of infection |
|  | Neighbors of vascular injury |
|  | Neighbors of preeclampsia |
|  | Neighbors of macrophage infiltration |
|  | Neighbors of liver injury |
|  | Neighbors of sepsis |
|  | Neighbors of death |
|  | Neighbors of autoimmune disease |
|  | Neighbors of ischemic stroke |
|  | Neighbors of dermatitis |
|  | Neighbors of heart disease |
|  | Neighbors of lung injury |
|  | Neighbors of immunopathology |
|  | Neighbors of systemic sclerosis |
|  | Neighbors of hepatocellular carcinoma |
|  | Neighbors of gastric cancer |
|  | Neighbors of atherosclerotic plaque |
|  | Neighbors of colorectal cancer |
|  | Neighbors of cardiac remodeling |
|  | Neighbors of breast cancer |
|  | Neighbors of melanoma |
|  | Neighbors of cartilage degeneration |
|  | Neighbors of interstitial fibrosis |
|  | Neighbors of neoplasm invasion |
|  | Neighbors of heart failure |
|  | Neighbors of pulmonary fibrosis |
|  | Neighbors of cancer |
|  | Neighbors of type 2 diabetes |
|  | Neighbors of neoplasm |
|  | Neighbors of endometriosis |
|  | Neighbors of myocardial fibrosis |
|  | Neighbors of coronary artery disease |
|  | Neighbors of rupture |
|  | Neighbors of prostate cancer |
|  | Neighbors of hypertrophy |
|  | Neighbors of osteolysis |
|  | Neighbors of ovarian cancer |
|  | Neighbors of pancreatic cancer |
|  | Neighbors of hypertension |
|  | Neighbors of epithelial cell |
|  | Neighbors of keratinocyte |
|  | Neighbors of vascular smooth muscle cell |
|  | Neighbors of umbilical vein endothelial cell |
|  | Neighbors of monocyte |
|  | Neighbors of endometrial stromal cell |
|  | Neighbors of alveolar macrophage |
|  | Neighbors of synoviocyte |
|  | Neighbors of macrophage |
|  | Neighbors of adipocyte |
|  | Neighbors of activated monocyte |
|  | Neighbors of fibroblast |
|  | Neighbors of airway epithelial cell |
|  | Neighbors of PBMC |
|  | Neighbors of stromal cell |
|  | Neighbors of airway smooth muscle cell |
|  | Neighbors of osteoblast |
|  | Neighbors of pneumocyte |
|  | Neighbors of endometrium cell |
|  | Neighbors of microglia |
|  | Neighbors of trophoblast |
|  | Neighbors of mononuclear cell |
|  | Neighbors of hepatic stellate cell |
|  | Neighbors of hepatocyte |
|  | Neighbors of decidual cell |
|  | Neighbors of kidney mesangial cell |
|  | Neighbors of endothelial cell |
|  | Neighbors of dendritic cell |
|  | Neighbors of Kuppfer cell |
|  | Neighbors of proadipocyte |
|  | Neighbors of inflammatory cell |
|  | Neighbors of osteoclast |
|  | Neighbors of neutrophil |
|  | Neighbors of smooth muscle myocyte |
|  | Neighbors of astrocyte |
|  | Neighbors of lung fibroblast |
|  | Neighbors of mesothelial cell |
|  | Neighbors of mononuclear phagocyte |
|  | Neighbors of capillary endothelial cell |
|  | Neighbors of granulocyte |
|  | Neighbors of myeloid cell |
|  | Neighbors of mesenchymal stroma cell |
|  | Neighbors of corneal epithelial cell |
|  | Neighbors of stem cell |
|  | Neighbors of tumor infiltrating macrophage |
|  | Neighbors of granulosa cell |
|  | Neighbors of leukocyte |
|  | Neighbors of chondrocyte |
|  | Neighbors of mesenchymal cell |
|  | Neighbors of T-cell |
|  | Neighbors of M1 macrophage |
|  | Neighbors of myofibroblast |
|  | Neighbors of mast cell |
|  | Neighbors of bone marrow stromal cell |
|  | Neighbors of aortic smooth muscle cell |
|  | Neighbors of mammary epithelial cell |
|  | Neighbors of phagocyte |
|  | Neighbors of kidney cell |
|  | Neighbors of M2 macrophage |
|  | Neighbors of blood cell |
|  | Neighbors of skin cell |
|  | Neighbors of luteal cell |
|  | Neighbors of lung |
|  | Neighbors of liver |
|  | Neighbors of skin |
|  | Neighbors of lymph node |
|  | Neighbors of airway |
|  | Neighbors of aorta |
|  | Neighbors of bone |
|  | Neighbors of carotid artery |
|  | Neighbors of synovial membrane |
|  | Neighbors of esophagus |
|  | Neighbors of retina |
|  | Neighbors of brain |
|  | Neighbors of ovary |
|  | Neighbors of breast |
|  | Neighbors of stomach |
|  | Neighbors of pancreas |
|  | Neighbors of lymphatic system |
|  | Neighbors of colorectal region |
|  | Neighbors of allantois |
|  | Neighbors of uterine cervix |
|  | Neighbors of nose |
|  | Neighbors of eye |
|  | Neighbors of cornea |
|  | Neighbors of periodontal ligament |
|  | Neighbors of hip region |
|  | Neighbors of NF-kB family |
|  | Neighbors of oxidized LDL |
|  | Neighbors of JNK |
|  | Neighbors of IL1 family |
|  | Neighbors of PPAR |
|  | Neighbors of Jun/Fos |
|  | Neighbors of NFKBI |
|  | Neighbors of PKC |
|  | Neighbors of PI3K |
|  | Neighbors of STAT family |
|  | Neighbors of mitogen-activated protein kinase |
|  | Neighbors of IFNAR ligand |
|  | Neighbors of transforming growth factor |
|  | Neighbors of TLR |
|  | Neighbors of cytokine |
|  | Neighbors of interferon |
|  | Neighbors of interleukin |
|  | Neighbors of JAK |
|  | Neighbors of MEK1/2 |
|  | Neighbors of PDGF |
|  | Neighbors of Ras GTPase |
|  | Neighbors of ERK1/2 |
|  | Neighbors of growth factor |
|  | Neighbors of PKA |
|  | Neighbors of ovalbumin |
|  | Neighbors of LDL |
|  | Neighbors of NF-AT family |
|  | Neighbors of chorionic gonadotropin |
|  | Neighbors of adipokine |
|  | Neighbors of protein tyrosine kinase |
|  | Neighbors of NAD(P)H oxidase |
|  | Neighbors of SMAD subfamily |
|  | Neighbors of AMPK |
|  | Neighbors of HDL |
|  | Neighbors of p38 |
|  | Neighbors of inflammatory cytokine |
|  | Neighbors of IL23 |
|  | Neighbors of estrogen receptor |
|  | Neighbors of histone deacetylase |
|  | Neighbors of PDGF-BB |
|  | Neighbors of IL1R |
|  | Neighbors of HIF-1 |
|  | Neighbors of IL12 |
|  | Neighbors of Notch |
|  | Neighbors of prostaglandin-endoperoxide synthase |
|  | Neighbors of mitogen-activated protein kinase kinase |
|  | Neighbors of TSH |
|  | Neighbors of endothelin |
|  | Neighbors of NOS |
|  | Neighbors of superoxide dismutase |
|  | Neighbors of proteasome endopeptidase complex |
|  | Neighbors of class nuclear receptor with C4 zinc fingers |
|  | Neighbors of histone H3 |
|  | Neighbors of vitronectin receptor (integrin) |
|  | Neighbors of PP2A |
|  | Neighbors of retinoid-X receptor subfamily |
|  | Neighbors of SAPK |
|  | Neighbors of WNT |
|  | Neighbors of gonadotropins |
|  | Neighbors of immunoglobulin |
|  | Neighbors of AHR subfamily |
|  | Neighbors of ITG |
|  | Neighbors of relaxin |
|  | Neighbors of TLR1/2 heterodimer |
|  | Neighbors of FSH |
|  | Neighbors of angiotensin receptor |
|  | Neighbors of RA receptor |
|  | Neighbors of Rho kinase |
|  | Neighbors of PDGF receptor |
|  | Neighbors of neuropeptides |
|  | Neighbors of histone acetyltransferase |
|  | 2\_Interleukin-17 and interleukin-22 signaling in psoriasis |
|  | 1\_TNF and IL1B provoke ECM degradation in osteoarthritis |
|  | 2\_Oxidized LDL causes endothelial dysfunction in atherosclerosis |
|  | 1\_Cigarette smoke causes dysfunction and production of inflammatory mediators in alveolar epithelial cells |
|  | 1\_2\_Feedback relations between coagulation and fibrinolysis |
|  | drug-target moltransport |
|  | Interleukin-17 and interleukin-22 signaling in psoriasis |

---

|  |  |
| --- | --- |
| MedScan ID | 4312 |

---

|  |  |
| --- | --- |
| LocusLink ID | 4312 |
|  | 83995 |
|  | 300339 |

---

|  |  |
| --- | --- |
| Alias | collagenase-like A |
|  | Mmp I |
|  | matrix metalloproteinase 1a (interstitial collagenase) |
|  | Interstitial collagenase |
|  | mammalian collagenase |
|  | Mmp1a |
|  | Mcola |
|  | matrix metallopeptidase 1a |
|  | 3.4.24.7 |
|  | myocardial collagenase |
|  | vertebrate collagenases |
|  | Matrix metalloproteinase-1 |
|  | matrix metalloproteinase 1 (interstitial collagenase) |
|  | matrix metalloproteinase-1a |
|  | matrix metallopeptidase 1a (interstitial collagenase) |
|  | LOC115169 |
|  | matrix metalloprotease 1 (interstitial collagenase) |
|  | Fibroblast collagenase |
|  | Matrix metalloprotease-1 |
|  | Matrix metalloproteinase I |
|  | matrix metallopeptidase 1 |
|  | LOC116389 |
|  | MM(01) |
|  | collagenase-like B |
|  | matrix metalloprotease 1a (interstitial collagenase) |
|  | MMP1 |
|  | matrix metallopeptidase 1 (interstitial collagenase) |
|  | Mcol-A |
|  | vertebrate collagenase |
|  | Matrix metalloprotease I |
|  | interstitial collagenase A |
|  | MM01\_HUMAN |
|  | CLG |
|  | CLGN |
|  | matrix metalloprotease 1 |
|  | matrix metalloproteinase 1 |
|  | MMP-1a |
|  | MMP-1 |

---

|  |  |
| --- | --- |
| EC Number | 3.4.24.7 |

---

|  |  |
| --- | --- |
| GO ID | 0004175 |
|  | 0004222 |
|  | 0004252 |
|  | 0008270 |
|  | 0044267 |
|  | 0030574 |
|  | 0019221 |
|  | 0022617 |
|  | 0030198 |
|  | 0050900 |
|  | 0032461 |
|  | 0006508 |
|  | 0016032 |
|  | 0031012 |
|  | 0005576 |
|  | 0005615 |
|  | 0008237 |
|  | 0008233 |
|  | 0071312 |
|  | 0035690 |
|  | 0071498 |
|  | 0071347 |
|  | 0071283 |
|  | 0071222 |
|  | 0071407 |
|  | 0071375 |
|  | 0071356 |
|  | 0071305 |
|  | 0001553 |
|  | 0001554 |
|  | 0010763 |
|  | 0014911 |
|  | 0042493 |
|  | 0014854 |
|  | 0071680 |
|  | 0009612 |
|  | 0005737 |
|  | 0005509 |
|  | 0005578 |
|  | 0016787 |
|  | 0046872 |
|  | 0007596 |

---

|  |  |
| --- | --- |
| KEGG ID | hsa:4312 |
|  | mmu:83995 |

---

|  |  |
| --- | --- |
| Organism | Homo sapiens {Organism urn:agi-taxid:9606} |
|  | Mus musculus {Organism urn:agi-taxid:10090} |
|  | Rattus norvegicus {Organism urn:agi-taxid:10116} |
|  | Homo sapiens |
|  | Mus musculus |
|  | Rattus norvegicus |

---

|  |  |
| --- | --- |
| Mouse chromosome position | 9 |

---

|  |  |
| --- | --- |
| OMIM ID | 120353 |
|  | 606963 |
|  | 226600 |

---

|  |  |
| --- | --- |
| Rat chromosome position | 8q11 |

---

|  |  |
| --- | --- |
| Hugo ID | 7155 |
|  | HGNC:7155 |

---

|  |  |
| --- | --- |
| Human chromosome position | 11q22.2 |
|  | 11q22.3 |

---

|  |  |
| --- | --- |
| Swiss-Prot Accession | B4DN15 |
|  | P03956 |
|  | Q53G95 |
|  | P03956.3 |
|  | Q9EPL5 |
|  | Q9EPL5.1 |
|  | B5DFD5 |
|  | P81563.1 |
|  | P08156 |
|  | Q149J4 |

---

|  |  |
| --- | --- |
| PIR ID | A37308 |

---

|  |  |
| --- | --- |
| GenBank ID | NC\_000011 |
|  | NM\_001145938 |
|  | NP\_001139410 |
|  | NM\_002421 |
|  | NP\_002412 |
|  | NG\_011740 |
|  | AF007878 |
|  | AF023338 |
|  | AJ002550 |
|  | AP000619 |
|  | AY769434 |
|  | AAV28732 |
|  | CH471065 |
|  | EAW67031 |
|  | HF583680 |
|  | CCQ43177 |
|  | M16567 |
|  | AAA52033 |
|  | U78045 |
|  | AAB36941 |
|  | AK024818 |
|  | AK098450 |
|  | AK130687 |
|  | AK223034 |
|  | BAD96754 |
|  | AK223035 |
|  | BAD96755 |
|  | AK223036 |
|  | BAD96756 |
|  | AK223056 |
|  | BAD96776 |
|  | AK223281 |
|  | BAD97001 |
|  | AK225495 |
|  | AK297723 |
|  | BAG60077 |
|  | AK301340 |
|  | BAG62888 |
|  | BC013118 |
|  | AAH13118 |
|  | BC013875 |
|  | AAH13875 |
|  | BT006874 |
|  | AAP35520 |
|  | BT020147 |
|  | AAV38949 |
|  | DC385245 |
|  | DQ399597 |
|  | ABD92796 |
|  | M13509 |
|  | AAA35699 |
|  | M15996 |
|  | AAA35700 |
|  | X05231 |
|  | CAA28858 |
|  | X54925 |
|  | CAA38691 |
|  | P03956 |
|  | NC\_000075 |
|  | NM\_032006 |
|  | NP\_114395 |
|  | AC115689 |
|  | CH467335 |
|  | EDL00990 |
|  | AJ278462 |
|  | CAC18880 |
|  | AK049552 |
|  | BAC33807 |
|  | BC117756 |
|  | AAI17757 |
|  | BQ558168 |
|  | Q9EPL5 |
|  | NC\_005107 |
|  | NM\_001134530 |
|  | NP\_001128002 |
|  | AC\_000076 |
|  | AABR07069019 |
|  | AAHX01053076 |
|  | BC169019 |
|  | AAI69019 |
|  | P81563 |
|  | NC\_018922 |
|  | AMYH02025488 |
|  | DQ890729 |
|  | ABM81655 |
|  | DQ893904 |
|  | ABM84830 |
|  | NW\_001034521 |
|  | AAHY01195788 |
|  | AAHY01195789 |
|  | AC\_000143 |
|  | ABBA01039931 |

---

|  |  |
| --- | --- |
| Swiss-Prot ID | MMP1\_HUMAN |
|  | MMP1A\_MOUSE |

---

|  |  |
| --- | --- |
| Cell Localization | Secreted |
|  | Extracellular space |
|  | Extracellular matrix |
|  | Secreted, extracellular space, extracellular matrix |

---

|  |  |
| --- | --- |
| Ensembl ID | ENSG00000196611 |
|  | ENSP00000322788.6 |
|  | ENST00000315274.7 |
|  | ENSMUSG00000043089 |
|  | ENSMUSP00000151193.1 |
|  | ENSMUST00000217651.1 |
|  | ENSRNOG00000032353 |
|  | ENSRNOP00000012595.2 |
|  | ENSRNOT00000012595.3 |
|  | ENSP00000322788 |
|  | ENST00000315274 |
|  | ENSMUSP00000034492 |
|  | ENSMUST00000034492 |
|  | ENSRNOP00000012595 |
|  | ENSRNOT00000012595 |

---

|  |  |
| --- | --- |
| MGI ID | MGI:1933846 |

---

|  |  |
| --- | --- |
| RGD ID | 1307917 |

---

|  |  |
| --- | --- |
| Unigene ID | Hs.83169 |
|  | Mm.156952 |
|  | Rn.79007 |

---

|  |  |
| --- | --- |
| Homologene ID | 20544 |

---
